# Exploring the NT-proBNP expression in Premature Infants with Patent Ductus Arteriosus (PDA) by Echocardiography

**DOI:** 10.12669/pjms.37.6-WIT.4853

**Published:** 2021

**Authors:** Yunlong Shi, Jianwei Ji, Chunying Wang

**Affiliations:** 1 Yunlong Shi, Attending Physician. Department of Neonatology, Yiwu Central Hospital, No. 519 Nanmen Road, Yiwu City, Zhejiang Province, 322000, China; 2 Jianwei Ji, Attending Physician. Department of Neonatology, Yiwu Central Hospital, No. 519 Nanmen Road, Yiwu City, Zhejiang Province, 322000, China; 3 Chunying Wang, Attending Physician. Department of Neonatology, Yiwu Central Hospital, No. 519 Nanmen Road, Yiwu City, Zhejiang Province, 322000, China

**Keywords:** Echocardiography, NT-proBNP, Symptomatic PDA

## Abstract

**Objectives:**

To investigate the correlation between echocardiographic indicators and the expression level of N-terminal pro-brain natriuretic peptide (NT-proBNP) in premature infants (PIs) with patent ductus arteriosus (PDA) and the value of NT-proBNP in diagnosing symptomatic PDA (sPDA) in PIs whose gestational age (GA) was less than 32 weeks.

**Methods:**

Ninty premature infants were selected as the research objects, including 52 in the non-PDA group and 38 in the PDA group (26 sPDA cases and 12 cases with asymptomatic PDA (asPDA)) from February 2019 to March 2020. The general information of these infants was recorded, including gender, delivery method, maternal infection, and serum NT-proBNP level on the 3rd day after birth. They were screened by echocardiographic indicators under an artificial intelligence convolutional neural network (AI-CNN). The Receiver Operating Characteristic (ROC) curves were illustrated to decide serum NT-proBNP expression levels, thereby determining specificity and sensitivity of sPDA and the correlation between serum sPDA NT-proBNP expression and echocardiographic indicators.

**Results:**

The expression level of serum NT-proBNP in the sPDA group was greater than that in the asPDA group and the non-PDA group (P<0.001). The serum NT-proBNP expression level was positively correlated with the diameter of the ductus arteriosus in the sPDA group (r=0.462, P<0.001); it was also positively correlated with the ratio of left atrium/aorta (LA/AO) (r=0.573, P<0.001), but was not correlated with left ventricular ejection fraction (LVEF) (r=-0.015, P=0.747).

**Conclusion:**

The combination of serum NT-proBNP expression and echocardiography had clinical values in early diagnosis of PDA.

## INTRODUCTION

Plasma N-terminal pro-brain natriuretic peptide (NT-proBNP) can reflect left ventricular load and pulmonary blood volume, and has the physiological functions of diuresis, natriuretic excretion, vasodilation, and reduction of peripheral vascular resistance.^1^-^4^ It can effectively regulate the changes in peripheral blood of cells and plays a very important role in the prediction of sPDA.^5^-^7^ The research results of PDA and NT-proBNP are different by many researchers, and there are differences due to various detection methods and observation populations.^8^-^10^ In this study, artificial intelligence convolutional neural network (AI-CNN) was adopted to optimize the echocardiography and improve image resolution, so as to study and analyze the ratio of left atrium/aorta (LA/AO) correlation, providing reference value for early diagnosis of PDA in clinic.^11^,^12^

## METHODS

From February 2019 to March 2020, 90 PIs born in our hospital were included as the research objects, including 38 PDA and 52 non-PDA infants. Of the 38 PDA infants, 26 were sPDA cases and 12 were asymptomatic PDA (asPDA) cases. The experiment had been submitted to the approval of the ethics committee of the hospital. Parents of the included infants had signed informed consent forms. Inclusion criteria: infants whose GA was between [28 weeks, 34 weeks]; infants whose body weight was between [1kg, 1.5kg]; infants whose admission to hospital was within 24 hours of birth. Exclusion criteria: infants with other congenital diseases; infants with septicemia or problems with liver and kidney function after one week of birth; infants whose information was incomplete.

The regressive analysis was used to collect medical records of PIs. The GA, delivery methods, gender, birth weight, maternal infection during pregnancy, premature rupture of membranes, and positive pressure ventilation of research objects were recorded. Also, the NT-proBNP levels within three days of the birth were also recorded, as well as the NT-proBNP levels in patients with sPDA with asPDA.

### NT-proBNP detection

For each included PI, 1mL of the peripheral venous blood sample was taken thre days after birth. The samples were stored in a sterile blood collection tube and sent to the laboratory for NT-proBNP detection. They were centrifuged at 3200 rpm for five minutes. The supernatant was pipetted into sterile tubes in an abacterial environment for NT-proBNP expression determination. If NT-proBNP test cannot be performed timely, the samples should be stored in a 4°C refrigerator (Qingdao Haier Group, China). Patients diagnosed with sPDA would be reviewed for NT-proBNP expression 72 hours after treatment.

The detection was performed by the principle of electrochemical luminescence and sandwich methods, and the measurement time was 20min. The instrument would automatically detect the concentration of the analyte in each sample (pmol/L or pg/mL). The conversion unit was pg/mL=pmol/L×8.457 and pmol/L=pg/mL×0.118. Even if the patient had jaundice (bilirubin < 428 umol/L) or hemolysis (Hb < 1.0 g/L), the test of NT-proBNP expression would not be affected. The detection instrument was a Cobase 601 electrochemical luminescence automatic immunoanalyzer (Roche, Switzerland). The detection kit was a brain natriuretic peptide precursor detection kit (Shanghai Roche Diagnostic Products, China).

### Echocardiography technology based on AI-CNN

CNN learns by local perception, and its neurons can use local to global methods to learn, just as humans learn from the outside. The information collected by the network from local regions can be used to learn the features of other regions. The only limit to the output of convolution values is the size of the convolution kernel, which is not related to other pixels. The image segmentation function layer is: (1) The input layer pre-processes the imported data information. (2) The convolution layer extracts the imported data information for feature extraction. If the input is N, the convolution kernel is m, the step size is v, and the edge zero-fill value is k, the size of the output feature map will be (N-m+2k)/v+1. (3) The excitation layer uses the sigmoid and RELU activation functions to perform nonlinear mapping analysis on the output of the convolution layer. (4) The pooling layer compresses the image data and parameters to reduce the number of overfittings. (5) The fully-connected layer: all neurons in adjacent layers are connected by weights at the end of CNN. (6) The classification layer: logistic regression and Softmax regression classification functions are utilized to classify the data information.

The PHILIPS iE Elite Color Doppler ultrasound system (Purchased from Shanghai Huanxi Medical Equipment, China) was used for routine inspection with a 3-5MHz probe. The included PIs received echocardiographic examinations to determine the presence of ductus arteriosus. Also, echocardiography was performed to the included PIs 72 hours after birth to detect the diameter of the ductus arteriosus, LA/AO ratio, and left ventricular ejection fraction (LVEF), thereby diagnosing the sPDA.

### Statistics Analysis

SPSS 26.0 software was used for data analysis. Data in normal distribution were written as χ ± s. The comparison between the two groups was tested by the t-test. The groups were compared pairwise by the Wilcoxon rank-sum test. The count data were written as frequency and rate. The two groups were compared by the χ^2^ test. All statistically significant differences were expressed as *P*<0.05.

## RESULTS

Ninety PIs were included, with 38 infants in the PDA group and 52 infants in the non-PDA group. The GA, delivery methods, gender, birth weight, maternal infection during pregnancy, premature rupture of membranes, and positive pressure ventilation of PIs were compared.

More patients with hemoptysis and hoarseness in pulmonary tuberculosis combined with lung cancer group than in the pulmonary tuberculosis group (*P*<0.05), and other symptoms were not significantly different (*P*>0.05) (Table-I).

**Table-I:** General information about PDA patients and infants without PDA.

*Data characteristics*		*PDA*	*Non-PDA*	*t/χ^2^*	**P**
Gender	Male	20	27	0.211	0.789
	Female	18	25		
Delivery methods	Cesarean section	20	26	0.014	0.868
	Spontaneous delivery	18	26		
GA (weeks)		31.5±1.2	31.7±1.1	-0.133	0.869
Birth weight (g)		1,496±241	1,543±262	-1.068	0.291
Maternal infection		1	4	1.514	0.264
Premature rupture of fetal membrane		5	9	0.631	0.501
Positive pressure ventilation		10	16	1.497	0.257

**Fig.1:**
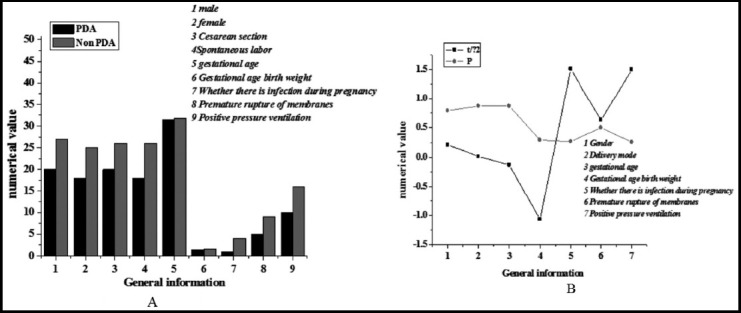
Comparison of general information between PDA patients and infants without PDA.

Fig.1 shows that there was no statistical significance in the number of PIs and the number of female PIs between the PDA group (28; 18) and the non-PDA group (27; 25) (*P*>0.05). The number of PIs delivered through the cesarean section in the PDA group and the non-PDA group was 20 and 26, respectively. The number of PIs delivered through spontaneous delivery in the PDA group (18) and the non-PDA group (26) was not statistically marked (*P*>0.05). The GA of PIs in the PDA group (31.5±1.2 weeks) and the non-PDA group (31.7±1.1 weeks) had no statistical significance (*P*>0.05). The birth weights of PIs in the PDA group and the non-PDA group were 1,496±241g and 1,543±262g in turn, and there was no statistical significance between the two groups (*P*>0.05). The number of maternal infections of PIs in the PDA group (1) was not statistically different from the number of the non-PDA group (4) (*P*>0.05). The numbers of premature rupture of membranes in the PDA group (5) and the non-PDA group (9) had no statistical significance (*P*>0.05). The number of positive pressure ventilation in the PDA group and the non-PDA group was 10 and 16, respectively, and there was no statistical significance between the two groups (*P*>0.05).

### NT-proBNP expression level comparison

The expression levels of NT-proBNP in infants of the four groups were measured (Table-II).

**Table-II:** The NT-proBNP expression levels of all PIs.

*Groups*	*NT-proBNP levels*	*Z*	**P**
PDA	12064 (7134,31498)	-9.897	0
Non-PDA	2498 (1399,3257)		
sPDA	12985 (7213,32011)	-10.101	0
asPDA	9089 (5903,25135)		

**Fig.2:**
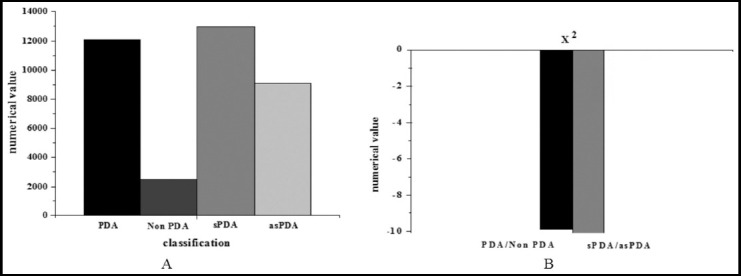
Comparison of NT-proBNP expression levels of PIs in each group.

Seventy two hours after the birth of PIs, NT-proBNP expression was greater in the PDA group than the non-PDA group, and greater in the sPDA group than the asPDA group (*P*<0.05). Fig-2. The Z value of NT-proBNP level comparison between the PDA group and the non-PDA group was -9.879, and Z value of NT-proBNP level comparison between the sPDA group and asPDA group was -10.101, without statistically significant differences.

**Fig.3:**
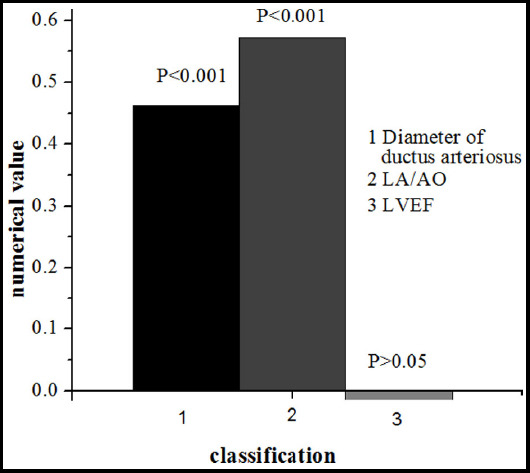
Correlation between NT-proBNP and echocardiographic indicators.

Echocardiography was performed on infants with sPDA at 72 hours after birth. The NT-proBNP expression levels were compared with the 3 echocardiographic indicators (Fig.3).

Seventy two hours after birth, serum NT-proBNP expression was positively correlated with the diameter of the ductus arteriosus on the 3rd day after birth in PIs with sPDA (r=0.462, *P*<0.001). It had positive correlation to left atrium/aorta (LA/AO) ratio (r=0.573, *P*<0.001) but no correlation to LVEF (r=-0.015, P=0.747).

## DISCUSSION

Studies have shown that NT-ProBNP has a sensitivity of 0.90 (95% CI: 0.79-0.96) and a specificity of 0.84 (96% CI: 0.77-0.90) for diagnosing hsPDA in preterm infants 72 hours after the birth of the newborn. Results of different diagnostic methods are quite different, which may also be related to the different diagnostic instruments and diagnostic methods. The results of this study showed that at 72 hours after birth, serum NT-proBNP expression in PI with sPDA was positively correlated with the diameter of the ductus arteriosus (r = 0.462, P < 0.001), and it ws positively correlated with the ratio of left atrium/aorta (LA/AO) (r = 0.573, P < 0.001), but was not correlated with LVEF.

If the weight of newborn < 1,000 g, the incidence of PDA is higher than 80%, hemodynamic stability is affected, and there will be some adverse symptoms, such as renal insufficiency, necrotizing enterocolitis, intraventricular hemorrhage, leading to neonatal mortality increasing by 4 - 8 times. NT-proBNP is produced by ventricular muscles under pressure or traction and secreted into the blood. Inflammatory factors will also affect. The ventricular volume of newborns is dilated and the volume load is false, which leads to the increase of physiological indicators of NT-ProBNP and the decrease of pulmonary vascular resistance, so that the ball filtration function is enhanced.^13^,^14^ The results of Kulkarni et al.^15^ showed that NT-ProBNP is different in the clinical diagnosis of hsPDA in PI, which may be related to the difference in measurement methods and instruments. It is recommended that the testing is performed according to local standardization to find the most suitable boundary for NT-proBNP. In this study, the serum NT-proBNP expression level of the sPDA group was higher than that of the asPDA group and the non-PDA group (P < 0.001), and the difference between the two groups was statistically obvious (P < 0.05). Some researchers have also compared the hsPDA group with the nhsPDA group and found that the younger the gestational age, the lower the birth weight of premature babies, the higher the probability of hs occurring; because PI has thinner arterial duct walls, lack of muscle tissue, and slower response to oxygen.^16^-^20^

## CONCLUSIONS

The expression levels of NT-proBNP were higher in PDA patients than those in non-PDA PIs; in the PDA group, the NT-proBNP levels were higher in sPDA patients than those in the asPDA patients. The level of serum NT-proBNP in infants with sPDA on the 3rd day after birth was positively correlated with the diameter of the ductus arteriosus and LA/AO. Therefore, serum NT-proBNP expression levels in PIs with sPDA significantly increased. The expression level of serum NT-proBNP on the 3rd day after birth was closely related to the diameter of ductus arteriosus and progress of LA/AO, which had clinical value for early diagnosis of PDA. However, the sample size of included PIs was small, which would lead to some errors or a bias compared to the actual situations. In the future, the sample size would be increased for in-depth research.

### Authors Contribution:

**YS:** Conceived the study, literature review, participated in its design, analyzed the data and helped to draft the manuscript, is responsible and accountable for the accuracy or integrity of the work. **CW:** Helped in design, data collection, article drafting & critical revision. **JJ:** Takes the responsibility and is accountable for all aspects of the work in ensuring that questions related to the accuracy or integrity of any part of the work are appropriately investigated and resolved.
